# Wood mouse body size measurements data in a Spanish protected area over two periods spanning thirty years

**DOI:** 10.1016/j.dib.2019.104024

**Published:** 2019-05-23

**Authors:** S. Santoro, M. Docampo, S. Moreno

**Affiliations:** a Department of Molecular Biology and Biochemical Engineering, University Pablo de Olavide, Carretera de Utrera Km. 1, 41013, Sevilla, Spain; b Ethology and Biodiversity Conservation Department, Doñana Biological Station-CSIC, Américo Vespucio 26, 41092, Seville, Spain

## Abstract

We present data of morphometric measurements of a wood mouse *Apodemus sylvaticus* population collected in the Doñana National Park (SW Spain) in the periods between 1978-81 and 2006-07. These data have been extrapolated from specimens deposited in the Doñana Biological Station Collection. The data in this article support the information provided in the research article “Marked reduction in body size of a wood mouse population in less than 30 years” [1].

**Table:** Specifications Table

Subject area	*Biology*
More specific subject area	*Ecology, Biology Conservation*
Type of data	*Figures and text file*
How data was acquired	*Data from Doñana Biological Station Collection*
Data format	*Analyzed*
Experimental factors	*The data proceed from specimens deposited in a Doñana Biological Station Collection (DBSC hereon) were collected from five zones within the Doñana National Park, all in the same biotope (scrublands). Detailed information on geographic coordinates is lacking.*
Experimental features	*The weight and the measurements of external trait as well as species and sex identification were recorded at the moment when the dead specimen was found in the trap. The cranial measurements were taken after the dissection in the laboratory of the Doñana Biological Station Collection. All length measurements were taken with a digital caliper to the nearest 0.*1 mm*.*
Data source location	*Doñana National Park (SW Spain).*
Data accessibility	*The data set is included in this article.*
Related research article	[1]*Docampo, M., Moreno, S., & Santoro, S. (2018). Marked reduction in body size of a wood mouse population in less than 30 years. Mammalian Biology.*

**Table:** 

**Value of the data** • Local and global processes provoke environmental changes in the Doñana area. Long-term data relative to populations' and individuals' traits of the Doñana species are needed to understand the causal processes beyond these changes. • They can be used as a reference to study morphological spatial and temporal changes in this and other mammal species. • They can promote further research to investigate the causes of the observed changes in the *Apodemus sylvaticus* body size. • They sum up to the growing literature about the historical reduction in body size observed in many animal species.

## 1 Data

This data set (Appendix S1) contains information of morphological measurements collated from dead specimens of the wood mouse *Apodemus sylvaticus* [2] that have been captured in the Doñana National Park (DNP). This protected area, located in South-West Spain, has undergone important vegetation changes in its recent history [3] (see Ref. [1] for details on the study site). The data proceed from 1026 individuals (416 females and 602 males) collected in 1978, 1980, 1981, 2006 and 2007. According to the information available in the DBSC about the sites of capture of the specimens, they all proceed from five zones within the DNP in the same biotope (scrublands). The skulls and skins of these specimens are deposited in the DBSC [4].

The weight and eight morphometric traits, four cranial and four external, have been measured. The cranial measurements are: Cranial Total Length (CTL), from the nasal end (rhinion) to the point of farthest occipital (ophistion); Condyle-Basal Length (CBL), from the proximal end of the occipital condyles to the pregnathion; Zygomatic Width (ZW), between the cheekbone salient points (zygion-zygion); and Diastema Length (DL). The external measurements are: Ear Length (EL), from the proximal sinus to the distal end; Foot Length (FL), from the back of the heel to the tip of the fingers; Tail Length (TL), from the tip to the base; and Head-Body Length (HBL).

**Fig. 1:**
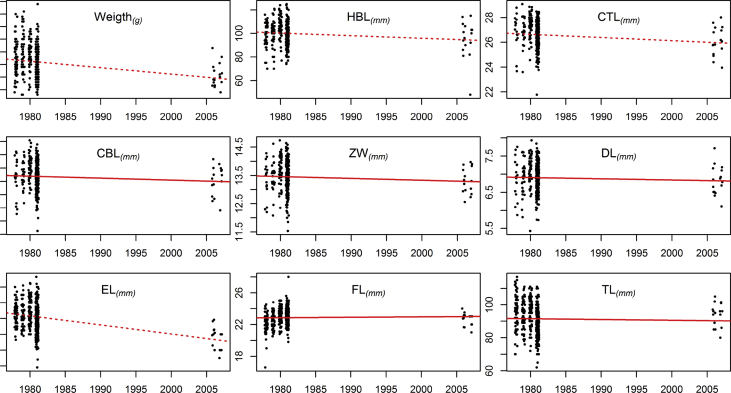
Scatter plots of *Apodemus sylvaticus* females' temporal (yearly) variation in weight and the eight morphological measurements. A dashed line indicates a statistically significant (p < 0.05) change (without controlling for any covariate).

**Fig. 2:**
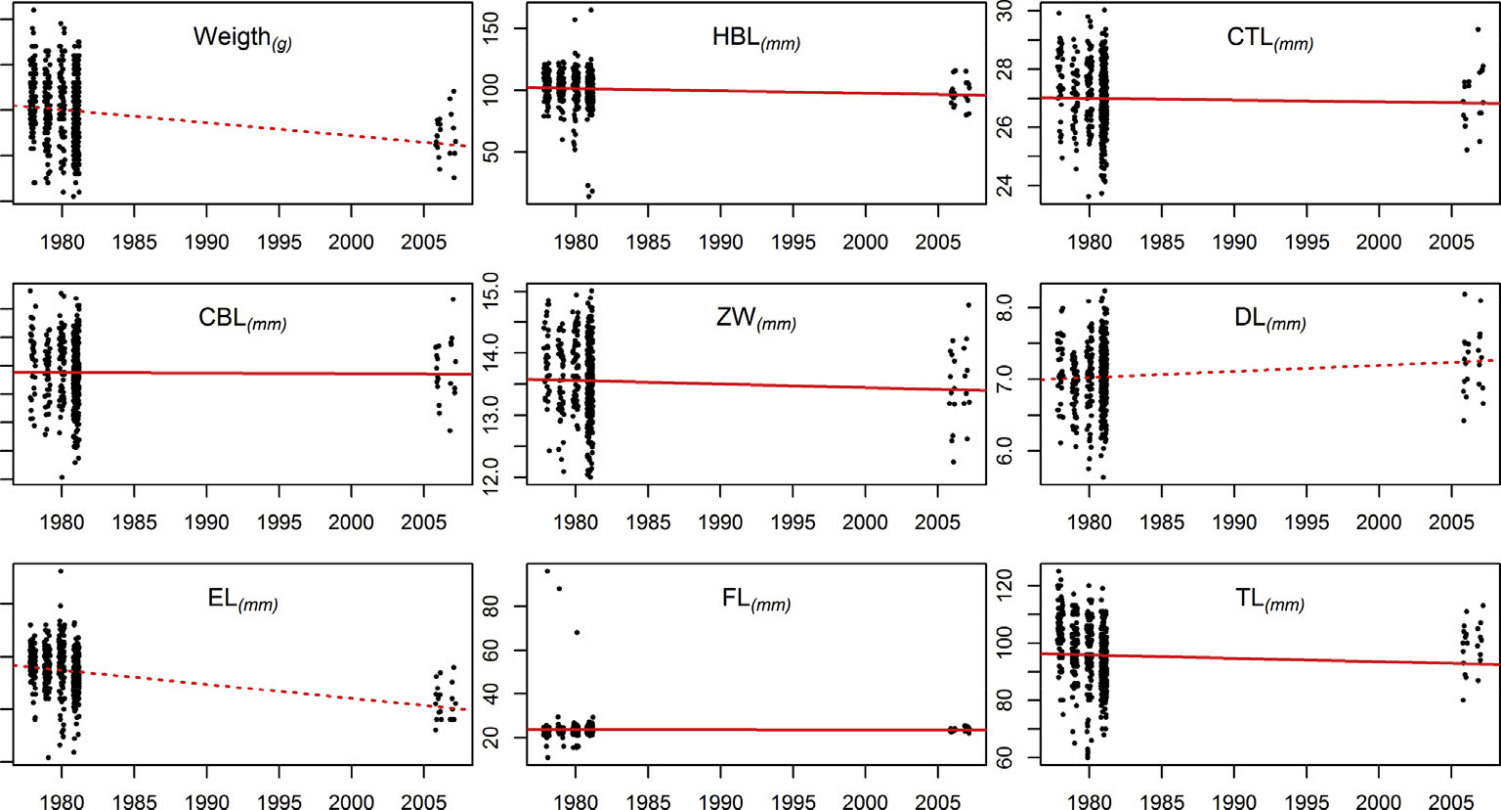
Scatter plots of *Apodemus sylvaticus* males' temporal (yearly) variation in weight and the eight morphological measurements. A dashed line indicates a statistically significant (p < 0.05) change (without controlling for any covariate).

A marked reduction in weight of the specimens between the periods 1979-81 and 2006-07 is evident both in females (Fig. 1A) and males (Fig. 2A). The other morphometric traits show heterogeneous patterns but most of them show a slight reduction (Fig. 1, Fig. 2). The showed changes are without controlling for individual and seasonal factors, for a detailed statistical analysis of these data see Ref. [1].

## 2 Experimental design, materials and methods

The data were collated from specimens preserved in the DBSC, where skins and skulls of the preserved specimens are deposited. The weight and the measurements of external trait as well as species and sex identification were recorded at the moment when the dead specimen was found in the trap (at DNP), before the dissection that was performed in the DBSC. Most of the collection specimens proceed from snap-traps (≈80%), that kill the mouse instantly, whereas others (≈16%), are specimens who died accidently in live-traps. This information was not available for the remaining portion of specimens (≈4%). Only the 3.1% of specimens have been captured in the second period (2006-07). This is a likely consequence of the major field-effort in the 1978-81 period but also of the decreasing abundance of the species in the area [1], [4], [5]. In the DBSC, all the morphometric data and the sex are annotated in a label together with the specimens’ skin and skull. The specimens without information on sex were discarded. In 2016, a further check of species identification was performed by Dra. S. Moreno on all the individuals recorded as *A. sylvaticus* in the DBSC whereas the cranial traits were measured. All length measurements were taken with a digital caliper to the nearest 0.1 mm. Individuals were classified into five age classes following Felten [6] criteria according to the degree of wearing of the upper molars assessed by visual examination with a stereoscopic micro-scope. The weight was measured by mean of a precision dynamometer (Pesola). Visual representation of the data (Fig. 1, Fig. 2) has been produced using R version 3.4.3 [7]. The R code used to load the data and reproduce the figures is available as Supplementary Material (Appendix S2).
