# Supplementary material for: Wood mouse body size measurements data in a Spanish protected area over two periods spanning thirty years
Source: Data Brief. 2019 May 23;25:104024. doi: 10.1016/j.dib.2019.104024 (PMC6586949; doi:10.1016/j.dib.2019.104024)
Supplement: Supplementary file 1 — Multimedia component 1 [file mmc1.docx]

The authors declare that they have no known competing financial interests or personal relationships that could have appeared to influence the work reported in this paper.

Simone Santoro on behalf of all authors.
